# Supplementary material for: Changes in the prevalence of self-reported sexually transmitted bacterial infections from 2010 and 2017 in two large European samples of men having sex with men–is it time to re-evaluate STI-screening as a control strategy?
Source: PLoS One. 2021 Mar 15;16(3):e0248582. doi: 10.1371/journal.pone.0248582 (PMC7959389; doi:10.1371/journal.pone.0248582)
Supplement: S1 Table — (DOCX) [file pone.0248582.s001.docx]

**S1 Table: Syphilis, self-reported diagnoses in the previous 12 months**

| **Country^1^** | **Total  N** | | **Syphilis diagnosis n (%)** | | Unclassifiable  n (%) | | Classified symptomatic n (%) | | Classified asymptomatic n (%) | | **Screened with a blood based STI test^2^ n/N (%)** | |
| --- | --- | --- | --- | --- | --- | --- | --- | --- | --- | --- | --- | --- |
|  | **2010** | **2017** | **2010** | **2017** | **2010** | **2017** | **2010** | **2017** | **2010** | **2017** | **2010** | **2017** |
| Albania/Montenegro/Kosovo**^‡^** | 102 | 171 | 2 (2.0) | 3 (1.8) | 1 (1) | 1 (0.6) | 1 (1.0) | 2 (1.2) | 0 (0) | 0 (0) | 11/100 (11.0) | 26/168 (15.5) |
| Austria | 4,195 | 2,705 | 95 (2.3) | 81 (3.0) | 50 (1.2) | 46 (1.7) | 25 (0.6) | 19 (0.7) | 20 (0.5) | 16 (0.6) | 729/4,120 (17.7) | 694/2,640 (26.3) |
| Belarus | 379 | 440 | 17 (4.5) | 8 (1.8) | 7 (1.9) | 6 (1.4) | 0 (0) | 0 (0) | 10 (2.6) | 2 (0.5) | 103/372 (27.7) | 144/434 (33.2) |
| Belgium | 4,129 | 3,038 | 129 (3.1) | 221 (7.3) | 69 (1.7) | 130 (4.3) | 44 (1.1) | 38 (1.3) | 16 (0.4) | 53 (1.7) | 1,187/4,016 (29.6) | 1,274/2,870 (44.4) |
| Bosnia & Herzegovina | 162 | 232 | 0 (0) | 0 (0) | 0 (0) | 0 (0) | 0 (0) | 0 (0) | 0 (0) | 0 (0) | 20/162 (12.3) | 41/232 (17.7) |
| Bulgaria | 1,078 | 1,177 | 12 (1.1) | 41 (3.5) | 6 (0.6) | 26 (2.2) | 4 (0.4) | 11 (0.9) | 2 (0.2) | 4 (0.3) | 203/1,068 (19.0) | 284/1,140 (24.9) |
| Croatia | 536 | 1,015 | 5 (0.9) | 11 (1.1) | 2 (0.4) | 9 (0.9) | 1 (0.2) | 1 (0.1) | 2 (0.4) | 1 (0.1) | 73/533 (13.7) | 245/1,005 (24.4) |
| Cyprus | 283 | 307 | 3 (1.1) | 10 (3.3) | 1 (0.4) | 3 (1.0) | 1 (0.4) | 3 (1.0) | 1 (0.4) | 4 (1.3) | 40/281 (14.2) | 109/301 (36.2) |
| Czech Republic | 2,491 | 1,897 | 40 (1.6) | 50 (2.6) | 21 (0.8) | 22 (1.2) | 14 (0.6) | 16 (0.8) | 5 (0.2) | 12 (0.6) | 282/2,456 (11.5) | 545/1,859 (29.3) |
| Denmark | 1,790 | 1,698 | 51 (2.9) | 52 (3.1) | 24 (1.3) | 26 (1.5) | 22 (1.2) | 16 (0.9) | 5 (0.3) | 10 (0.6) | 347/1,744 (19.9) | 553/1,656 (33.4) |
| Estonia | 605 | 212 | 4 (0.7) | 4 (1.9) | 2 (0.3) | 3 (1.4) | 1 (0.2) | 1 (0.5) | 1 (0.2) | 0 (0) | 77/602 (12.8) | 47/208 (22.6) |
| Finland | 2,061 | 1,409 | 13 (0.6) | 18 (1.3) | 4 (0.2) | 10 (0.7) | 3 (0.2) | 5 (0.4) | 6 (0.3) | 3 (0.2) | 310/2,054 (15.1) | 345/1,394 (24.7) |
| France* | 11,757 | 10,996 | 371 (3.2) | 1,399 (12.7) | 195 (1.7) | 612 (5.6) | 79 (0.7) | 160 (1.5) | 97 (0.8) | 627 (5.7) | 3,357/11,483 (29.2) | 4,553/10,224 (44.5) |
| Germany | 55,844 | 23,107 | 1,062 (1.9) | 753 (3.3) | 530 (1) | 456 (2.0) | 371 (0.7) | 179 (0.8) | 161 (0.3) | 118 (0.5) | 9,048/54,943 (16.5) | 5,748/22,472 (25.6) |
| Greece | 3,223 | 2,909 | 55 (1.7) | 99 (3.4) | 26 (0.8) | 52 (1.8) | 23 (0.7) | 35 (1.2) | 6 (0.2) | 12 (0.4) | 645/3,174 (20.3) | 812/2,822 (28.8) |
| Hungary | 2,131 | 2,177 | 42 (2.0) | 58 (2.7) | 26 (1.2) | 33 (1.5) | 7 (0.3) | 16 (0.7) | 9 (0.4) | 9 (0.4) | 362/2,098 (17.3) | 413/2,128 (19.4) |
| Iceland | 75 | 111 | 0 (0) | 3 (2.7) | 0 (0) | 3 (2.7) | 0 (0) | 0 (0) | 0 (0) | 0 (0) | 23/75 (30.7) | 46/108 (42.6) |
| Ireland | 2,289 | 2,083 | 52 (2.3) | 66 (3.2) | 33 (1.4) | 48 (2.3) | 11 (0.5) | 8 (0.4) | 8 (0.4) | 10 (0.5) | 604/2,245 (26.9) | 846/2,027 (41.7) |
| Italy* | 16,678 | 11,025 | 446 (2.7) | 405 (3.7) | 218 (1.3) | 216 (2.0) | 166 (1.0) | 126 (1.1) | 62 (0.4) | 63 (0.6) | 3,275/16,294 (20.1) | 2,872/10,683 (26.9) |
| Latvia | 723 | 252 | 7 (1.0) | 7 (2.8) | 3 (0.4) | 5 (2.0) | 2 (0.3) | 1 (0.4) | 2 (0.3) | 1 (0.4) | 103/718 (14.3) | 78/246 (31.7) |
| Lithuania | 614 | 370 | 1 (0.2) | 4 (1.1) | 1 (0.2) | 2 (0.5) | 0 (0) | 0 (0) | 0 (0) | 2 (0.5) | 52/613 (8.5) | 55/368 (14.9) |
| Luxembourg | 287 | 169 | 8 (2.8) | 3 (1.8) | 6 (2.1) | 3 (1.8) | 1 (0.4) | 0 (0) | 1 (0.4) | 0 (0) | 50/280 (17.9) | 62/166 (37.3) |
| Malta | 122 | 299 | 1 (0.8) | 9 (3.0) | 0 (0) | 4 (1.3) | 0 (0) | 2 (0.7) | 1 (0.8) | 3 (1.0) | 28/122 (23.0) | 108/293 (36.9) |
| Moldova | 123 | 498 | 4 (3.3) | 29 (5.8) | 2 (1.6) | 15 (3.0) | 0 (0) | 8 (1.6) | 2 (1.6) | 6 (1.2) | 29/121 (24.0) | 193/475 (40.6) |
| Netherlands | 3,912 | 3,851 | 127 (3.2) | 200 (5.2) | 78 (2.0) | 130 (3.4) | 35 (0.9) | 41 (1.1) | 14 (0.4) | 29 (0.8) | 1,382/3,799 (36.4) | 1,737/3,680 (47.2) |
| North Macedonia | 126 | 175 | 0 (0) | 1 (0.6) | 0 (0) | 0 (0) | 0 (0) | 1 (0.6) | 0 (0) | 0 (0) | 22/126 (17.5) | 34/174 (19.5) |
| Norway | 2,151 | 2,957 | 15 (0.7) | 44 (1.5) | 10 (0.5) | 30 (1.0) | 3 (0.1) | 11 (0.4) | 2 (0.1) | 3 (0.1) | 501/2,138 (23.4) | 997/2,916 (34.2) |
| Poland | 2,868 | 4,025 | 62 (2.2) | 173 (4.3) | 31 (1.1) | 101 (2.5) | 23 (0.8) | 52 (1.3) | 8 (0.3) | 20 (0.5) | 476/2,814 (16.9) | 957/3,872 (24.7) |
| Portugal | 5,386 | 2,555 | 179 (3.3) | 171 (6.7) | 88 (1.6) | 90 (3.5) | 68 (1.3) | 49 (1.9) | 23 (0.4) | 32 (1.3) | 922/5,230 (17.6) | 816/2,416 (33.8) |
| Romania | 2,451 | 2,002 | 47 (1.9) | 52 (2.6) | 29 (1.2) | 34 (1.7) | 17 (0.7) | 11 (0.6) | 1 (0) | 7 (0.4) | 528/2,405 (22.0) | 534/1,957 (27.3) |
| Russia | 5,258 | 6,247 | 251 (4.8) | 209 (3.4) | 124 (2.4) | 106 (1.7) | 32 (0.6) | 68 (1.1) | 95 (1.8) | 35 (0.6) | 1,351/5,102 (26.5) | 2,018/6,073 (33.2) |
| Serbia | 1,147 | 1,041 | 8 (0.7) | 24 (2.3) | 3 (0.3) | 14 (1.3) | 3 (0.3) | 8 (0.8) | 2 (0.2) | 2 (0.2) | 201/1,141 (17.6) | 176/1,019 (17.3) |
| Slovakia | 605 | 1,003 | 3 (0.5) | 12 (1.2) | 2 (0.3) | 8 (0.8) | 1 (0.2) | 2 (0.2) | 0 (0) | 2 (0.2) | 62/602 (10.3) | 168/993 (16.9) |
| Slovenia | 1,032 | 685 | 14 (1.4) | 17 (2.5) | 9 (0.9) | 5 (0.7) | 5 (0.5) | 8 (1.2) | 0 (0) | 4 (0.6) | 112/1,018 (11.0) | 252/672 (37.5) |
| Spain* | 13,730 | 10,652 | 515 (3.8) | 569 (5.3) | 240 (1.8) | 344 (3.2) | 190 (1.4) | 142 (1.3) | 85 (0.6) | 83 (0.8) | 3,081/13,300 (23.2) | 3,607/10,166 (35.5) |
| Sweden | 3,252 | 4,443 | 20 (0.6) | 53 (1.2) | 11 (0.3) | 34 (0.8) | 4 (0.1) | 12 (0.3) | 5 (0.2) | 7 (0.2) | 841/3,237 (26.0) | 1,187/4,397 (27.0) |
| Switzerland* | 5,180 | 3,383 | 118 (2.3) | 147 (4.4) | 58 (1.1) | 76 (2.3) | 43 (0.8) | 28 (0.8) | 17 (0.3) | 43 (1.3) | 982/5,079 (19.3) | 1,173/3,279 (35.8) |
| Turkey | 1,987 | 1,855 | 12 (0.6) | 51 (2.8) | 6 (0.3) | 31 (1.7) | 6 (0.3) | 13 (0.7) | 0 (0) | 7 (0.4) | 166/1,975 (8.4) | 459/1,811 (25.3) |
| Ukraine | 1,785 | 1,201 | 91 (5.1) | 28 (2.3) | 42 (2.4) | 12 (1.0) | 6 (0.3) | 10 (0.8) | 43 (2.4) | 6 (0.5) | 347/1,737 (20.0) | 366/1,179 (31.0) |
| United Kingdom | 18,432 | 11,889 | 379 (2.1) | 359 (3.0) | 242 (1.3) | 240 (2.0) | 89 (0.5) | 56 (0.5) | 48 (0.3) | 63 (0.5) | 5,370/18,101 (29.7) | 4,940/11,593 (42.6) |
| **Total** | **180,979** | **126,261** | **4,261 (2.4)** | **5,444 (4.3)** | **2,200 (1.2)** | **2,986 (2.4)** | **1,301 (0.7)** | **1,159 (0.9)** | **760 (0.4)** | **1,299 (1.0)** | **37,302/177,478(21.0)** | **39,514/122,116 (32.4) (32.4)** |

^1^ This study includes 46 countries, with four European microstates included in neighbouring (Andorra, Liechtenstein) or surrounding (Monaco, San Marino) countries, and with Albania, Montenegro and Kosovo merged to form a region; this results in 40 country-like entities included in this table. ^2^ Subtracted from numerator and denominator are men reporting syphilis that was unclassifiable or classified as symptomatic. *Including overseas territories/dependencies and/or a microstate. **^‡^**The designation of Kosovo is without prejudice to positions on status and is in line with UNSCR 1244/1999 and the International Court of Justice Opinion on the Kosovo declaration of independence.
