# Supplementary material for: Changes in the prevalence of self-reported sexually transmitted bacterial infections from 2010 and 2017 in two large European samples of men having sex with men–is it time to re-evaluate STI-screening as a control strategy?
Source: PLoS One. 2021 Mar 15;16(3):e0248582. doi: 10.1371/journal.pone.0248582 (PMC7959389; doi:10.1371/journal.pone.0248582)
Supplement: S2 Table — (DOCX) [file pone.0248582.s002.docx]

**S2 Table: Gonorrhoea/Chlamydia (Gon/Chl), self-reported diagnoses in the previous 12 months**

| **Country^1^** | **Total  N** | | **Gon/Chl diagnosis n (%)** | | Unclassifiable  n (%) | | Classified symptomatic n (%) | | Classified asymptomatic n (%) | | **Screened with a urine-based STI test^2^  (or genital swab) *and* anal swab; n/N (%)** | |
| --- | --- | --- | --- | --- | --- | --- | --- | --- | --- | --- | --- | --- |
|  | **2010** | **2017** | **2010** | **2017** | **2010** | **2017** | **2010** | **2017** | **2010** | **2017** | **2010** | **2017** |
| Albania/Montenegro/Kosovo**^‡^** | 102 | 171 | 1 (1.0) | 10 (5.8) | 1 (1.0) | 6 (3.5) | 0 (0) | 3 (1.8) | 0 (0) | 1 (0.6) | 1/101 (1.0) | 5/162 (3.1) |
| Austria | 4,195 | 2,705 | 148 (3.5) | 166 (6.1) | 63 (1.5) | 88 (3.3) | 62 (1.5) | 50 (1.9) | 23 (0.6) | 28 (1.0) | 149/4,070 (3.7) | 174/2,567 (6.8) |
| Belarus | 379 | 440 | 28 (7.4) | 21 (4.8) | 9 (2.4) | 7 (1.6) | 8 (2.1) | 10 (2.3) | 11 (2.9) | 4 (0.9) | 22/362 (6.1) | 33/423 (7.8) |
| Belgium | 4,129 | 3,038 | 247 (6.0) | 321 (10.6) | 137 (3.3) | 184 (6.1) | 85 (2.1) | 66 (2.2) | 25 (0.6) | 71 (2.3) | 60/3,907 (1.5) | 277/2,788 (9.9) |
| Bosnia & Herzegovina | 162 | 232 | 5 (3.1) | 4 (1.7) | 2 (1.2) | 1 (0.4) | 3 (1.9) | 2 (0.9) | 0 (0) | 1 (0.4) | 3/157 (1.9) | 6/229 (2.6) |
| Bulgaria | 1,078 | 1,177 | 29 (2.7) | 59 (5.0) | 16 (1.5) | 41 (3.5) | 10 (0.9) | 14 (1.2) | 3 (0.3) | 4 (0.3) | 22/1,052 (2.1) | 25/1,122 (2.2) |
| Croatia | 536 | 1,015 | 8 (1.5) | 38 (3.7) | 4 (0.8) | 21 (2.1) | 3 (0.6) | 12 (1.2) | 1 (0.2) | 5 (0.5) | 6/529 (1.1) | 42/982 (4.3) |
| Cyprus | 283 | 307 | 12 (4.2) | 18 (5.9) | 6 (2.1) | 10 (3.3) | 3 (1.1) | 7 (2.3) | 3 (1.1) | 1 (0.3) | 5/274 (1.8) | 10/290 (3.4) |
| Czech Republic | 2,491 | 1,897 | 63 (2.5) | 88 (4.6) | 35 (1.4) | 39 (2.1) | 21 (0.8) | 26 (1.4) | 7 (0.3) | 23 (1.2) | 42/2,435 (1.7) | 131/1,832 (7.2) |
| Denmark | 1,790 | 1,698 | 91 (5.1) | 176 (10.4) | 34 (1.9) | 84 (5.0) | 47 (2.6) | 63 (3.7) | 10 (0.6) | 29 (1.7) | 122/1,709 (7.1) | 235/1,551 (15.2) |
| Estonia | 605 | 212 | 12 (2.0) | 9 (4.2) | 7 (1.2) | 7 (3.3) | 2 (0.3) | 2 (0.9) | 3 (0.5) | 0 (0) | 14/596 (2.3) | 6/203 (3.0) |
| Finland | 2,061 | 1,409 | 47 (2.3) | 70 (5.0) | 17 (0.8) | 37 (2.6) | 19 (0.9) | 16 (1.1) | 11 (0.5) | 17 (1.2) | 117/2,025 (5.8) | 141/1,356 (10.4) |
| France* | 11,757 | 10,996 | 636 (5.4) | 1,766 (16.1) | 323 (2.8) | 848 (7.7) | 217 (1.9) | 331 (3.0) | 96 (0.8) | 587 (5.3) | 183/11,217 (1.6) | 1,293/9,817 (13.2) |
| Germany | 55,844 | 23,107 | 2,039 (3.7) | 1,526 (6.6) | 1,067 (1.9) | 803 (3.5) | 743 (1.3) | 465 (2.0) | 229 (0.4) | 258 (1.1) | 1,439/54,034 (2.7) | 1,667/21,839 (7.6) |
| Greece | 3,223 | 2,909 | 76 (2.4) | 82 (2.8) | 41 (1.3) | 47 (1.6) | 24 (0.7) | 26 (0.9) | 11 (0.3) | 9 (0.3) | 61/3,158 (1.9) | 65/2,836 (2.3) |
| Hungary | 2,131 | 2,177 | 47 (2.2) | 71 (3.3) | 23 (1.1) | 39 (1.8) | 15 (0.7) | 26 (1.2) | 9 (0.4) | 6 (0.3) | 43/2,093 (2.1) | 60/2,112 (2.8) |
| Iceland | 75 | 111 | 3 (4.0) | 11 (9.9) | 2 (2.7) | 7 (6.3) | 1 (1.3) | 2 (1.8) | 0 (0) | 2 (1.8) | 3/72 (4.2) | 23/102 (22.5) |
| Ireland | 2,289 | 2,083 | 118 (5.2) | 255 (12.2) | 47 (2.1) | 144 (6.9) | 46 (2.0) | 59 (2.8) | 25 (1.1) | 52 (2.5) | 425/2,196 (19.4) | 567/1,880 (30.2) |
| Italy* | 16,678 | 11,025 | 497 (3.0) | 374 (3.4) | 324 (1.9) | 233 (2.1) | 118 (0.7) | 91 (0.8) | 55 (0.3) | 50 (0.5) | 316/16,236 (1.9) | 345/10,701 (3.2) |
| Latvia | 723 | 252 | 11 (1.5) | 8 (3.2) | 2 (0.3) | 3 (1.2) | 7 (1.0) | 5 (2.0) | 2 (0.3) | 0 (0) | 15/714 (2.1) | 12/244 (4.9) |
| Lithuania | 614 | 370 | 10 (1.6) | 6 (1.6) | 5 (0.8) | 2 (0.5) | 2 (0.3) | 2 (0.5) | 3 (0.5) | 2 (0.5) | 9/607 (1.5) | 7/366 (1.9) |
| Luxembourg | 287 | 169 | 4 (1.4) | 10 (5.9) | 4 (1.4) | 7 (4.1) | 0 (0) | 1 (0.6) | 0 (0) | 2 (1.2) | 1/283 (0.4) | 10/161 (6.2) |
| Malta | 122 | 299 | 6 (4.8) | 2 (1.1) | 3 (2.4) | 2 (1.1) | 2 (1.6) | 0 (0) | 1 (0.8) | 0 (0) | 19/119 (16.0) | 80/277 (28.9) |
| Moldova | 123 | 498 | 3 (2.5) | 26 (8.7) | 1 (0.8) | 14 (4.7) | 2 (1.6) | 8 (2.7) | 0 (0) | 4 (1.3) | 4/119 (3.4) | 4/494 (0.8) |
| Netherlands | 3,912 | 3,851 | 397 (10.1) | 631 (16.4) | 182 (4.7) | 362 (9.4) | 139 (3.6) | 149 (3.9) | 76 (1.9) | 120 (3.1) | 822/3,591 (22.9) | 1,201/3,340 (36.0) |
| North Macedonia | 126 | 175 | 6 (4.9) | 6 (1.2) | 2 (1.6) | 2 (0.4) | 2 (1.6) | 0 (0) | 1 (0.8) | 0 (0) | 4/121 (3.3) | 4/173 (2.3) |
| Norway | 2,151 | 2,957 | 108 (5.0) | 228 (7.7) | 40 (1.9) | 130 (4.4) | 43 (2.0) | 66 (2.2) | 25 (1.2) | 32 (1.1) | 246/2,068 (11.9) | 570/2,761 (20.6) |
| Poland | 2,868 | 4,025 | 68 (2.4) | 146 (3.6) | 48 (1.7) | 86 (2.1) | 16 (0.6) | 46 (1.1) | 4 (0.1) | 14 (0.4) | 31/2,804 (1.1) | 59/3,893 (1.5) |
| Portugal | 5,386 | 2,555 | 188 (3.5) | 189 (7.4) | 121 (2.3) | 114 (4.5) | 46 (0.9) | 42 (1.6) | 21 (0.4) | 33 (1.3) | 76/5,219 (1.5) | 131/2,399 (5.5) |
| Romania | 2,451 | 2,002 | 68 (2.8) | 56 (2.8) | 46 (1.9) | 32 (1.6) | 18 (0.7) | 12 (0.6) | 4 (0.2) | 12 (0.6) | 29/2,387 (1.2) | 40/1,958 (2.0) |
| Russia | 5,258 | 6,247 | 305 (5.8) | 217 (3.5) | 137 (2.6) | 90 (1.4) | 80 (1.5) | 98 (1.6) | 88 (1.7) | 29 (0.5) | 296/5,041 (5.9) | 409/6,059 (6.8) |
| Serbia | 1,147 | 1,041 | 20 (1.7) | 18 (1.7) | 13 (1.1) | 14 (1.3) | 4 (0.4) | 3 (0.3) | 3 (0.3) | 1 (0.1) | 9/1,130 (0.8) | 15/1,024 (1.5) |
| Slovakia | 605 | 1,003 | 7 (1.2) | 23 (2.3) | 5 (0.8) | 11 (1.1) | 2 (0.3) | 7 (0.7) | 0 (0) | 5 (0.5) | 9/598 (1.5) | 24/985 (2.4) |
| Slovenia | 1,032 | 685 | 22 (2.1) | 23 (3.4) | 12 (1.2) | 14 (2.0) | 7 (0.7) | 3 (0.4) | 3 (0.3) | 6 (0.9) | 25/1,013 (2.5) | 47/668 (7.0) |
| Spain* | 13,730 | 10,652 | 695 (5.1) | 874 (8.2) | 345 (2.5) | 451 (4.2) | 262 (1.9) | 286 (2.7) | 88 (0.6) | 137 (1.3) | 307/13,123 (2.3) | 684/9,915 (6.9) |
| Sweden | 3,252 | 4,443 | 141 (4.3) | 268 (6.0) | 47 (1.5) | 144 (3.2) | 65 (2.0) | 60 (1.4) | 29 (0.9) | 64 (1.4) | 522/3,140 (16.6) | 708/4,239 (16.7) |
| Switzerland* | 5,180 | 3,383 | 258 (5.0) | 321 (9.5) | 111 (2.1) | 155 (4.6) | 115 (2.2) | 84 (2.5) | 32 (0.6) | 82 (2.4) | 114/4,954 (2.3) | 430/3,144 (13.7) |
| Turkey | 1,987 | 1,855 | 107 (5.4) | 124 (6.7) | 75 (3.8) | 85 (4.6) | 28 (1.4) | 18 (1.0) | 4 (0.2) | 21 (1.1) | 24/1,884 (1.3) | 28/1,752 (1.6) |
| Ukraine | 1,785 | 1,201 | 95 (5.3) | 40 (3.3) | 48 (2.7) | 15 (1.3) | 22 (1.2) | 21 (1.8) | 25 (1.4) | 4 (0.3) | 59/1,715 (3.4) | 46/1,165 (3.9) |
| United Kingdom | 18,432 | 11,889 | 1,290 (7.0) | 1,399 (11.8) | 581 (3.2) | 776 (6.5) | 449 (2.4) | 321 (2.7) | 260 (1.4) | 302 (2.5) | 3,854/17,402 (22.1) | 3,511/10,792 (32.5) |
| **Total** | **180,979** | **126,261** | **7,916 (4.4)** | **9,680 (7.7)** | **3,986 (2.2)** | **5,155 (4.1)** | **2,738 (1.5)** | **2,505 (2.0)** | **1,192 (0.7)** | **2,020 (1.6)** | **9,508/174,255 (5.5)** | **13,125/118,601 (11.1)** |

^1^ This study includes 46 countries, with four European microstates included in neighbouring (Andorra, Liechtenstein) or surrounding (Monaco, San Marino) countries, and with Albania, Montenegro and Kosovo merged to form a region; this results in 40 country-like entities included in this table. ^2^ Subtracted from numerator and denominator are men reporting gonorrhoea/chlamydia that was unclassifiable or classified as symptomatic. *Including overseas territories/dependencies and/or a microstate. **^‡^**The designation of Kosovo is without prejudice to positions on status and is in line with UNSCR 1244/1999 and the International Court of Justice Opinion on the Kosovo declaration of independence.
