# Supplementary material for: Changes in the prevalence of self-reported sexually transmitted bacterial infections from 2010 and 2017 in two large European samples of men having sex with men–is it time to re-evaluate STI-screening as a control strategy?
Source: PLoS One. 2021 Mar 15;16(3):e0248582. doi: 10.1371/journal.pone.0248582 (PMC7959389; doi:10.1371/journal.pone.0248582)
Supplement: S3 Table — (DOCX) [file pone.0248582.s003.docx]

**S3 Table: Partner numbers, condom use with the last non-steady partner, and STI screen in the previous six months**

| **Country^1^** | **Total N** | | **More than 10 partners n (%)** | | **CAI with last non-steady partner^2^ n/N (%)** | | **STI screen in the previous six months^2^ n/N (%)** | |
| --- | --- | --- | --- | --- | --- | --- | --- | --- |
|  | **2010** | **2017** | **2010** | **2017** | **2010** | **2017** | **2010** | **2017** |
| Albania/Montenegro/Kosovo**^‡^** | 102 | 171 | 24 (23.5) | 22 (12.9) | 18/87 (20.7) | 35/171 (20.5) | 6/95 (6.3) | 13/163 (8.0) |
| Austria | 4,195 | 2,705 | 1,009 (24.1) | 678 (25.1) | 495/3,619 (13.7) | 614/2,703 (22.7) | 557/3,847 (14.5) | 567/2,586 (21.9) |
| Belarus | 379 | 440 | 45 (11.9) | 76 (17.3) | 63/345 (18.3) | 81/439 (18.5) | 68/363 (18.7) | 113/431 (26.2) |
| Belgium | 4,129 | 3,038 | 1,333 (32.3) | 1,158 (38.1) | 495/3,529 (14.0) | 787/3,027 (26.0) | 925/3,907 (23.7) | 1,101/2,940 (37.4) |
| Bosnia & Herzegovina | 162 | 232 | 16 (9.9) | 29 (12.5) | 33/136 (24.3) | 51/230 (22.2) | 12/156 (7.7) | 37/219 (16.9) |
| Bulgaria | 1,078 | 1,177 | 227 (21.1) | 243 (20.7) | 150/927 (16.2) | 268/1,174 (22.8) | 136/983 (13.8) | 226/1,120 (20.2) |
| Croatia | 536 | 1,015 | 86 (16.0) | 131 (12.9) | 55/463 (11.9) | 230/1,007 (22.8) | 46/523 (8.8) | 197/982 (20.1) |
| Cyprus | 283 | 307 | 77 (27.2) | 86 (28.0) | 50/241 (20.7) | 59/307 (19.2) | 33/267 (12.4) | 89/292 (30.5) |
| Czech Republic | 2,491 | 1,897 | 346 (13.9) | 378 (19.9) | 283/2,079 (13.6) | 419/1,890 (22.2) | 204/2,304 (8.9) | 391/1,814 (21.6) |
| Denmark | 1,790 | 1,698 | 477 (26.7) | 526 (31.0) | 257/1,577 (16.3) | 504/1,692 (29.8) | 262/1,742 (15.0) | 435/1,657 (26.3) |
| Estonia | 605 | 212 | 84 (13.9) | 38 (17.9) | 82/518 (15.8) | 49/212 (23.1) | 55/573 (9.6) | 39/205 (19.0) |
| Finland | 2,061 | 1,409 | 332 (16.1) | 287 (20.4) | 205/1,735 (11.8) | 298/1,401 (21.3) | 200/2,009 (10.0) | 254/1,373 (18.5) |
| France* | 11,757 | 10,996 | 4,395 (37.4) | 4,668 (42.5) | 1,235/10,419 (11.9) | 3,054/10,960 (27.9) | 2,533/11,323 (22.4) | 4,091/10,677 (38.3) |
| Germany | 55,844 | 23,107 | 12,786 (22.9) | 5,993 (25.9) | 7,705/47,213 (16.3) | 5,907/23,027 (25.7) | 6,857/51,352 (13.4) | 4,632/22,018 (21.0) |
| Greece | 3,223 | 2,909 | 866 (26.9) | 817 (28.1) | 284/2,677 (10.6) | 485/2,896 (16.7) | 446/3,091 (14.4) | 621/2,809 (22.1) |
| Hungary | 2,131 | 2,177 | 390 (18.3) | 457 (21.0) | 261/1,841 (14.2) | 491/2,165 (22.7) | 275/2,052 (13.4) | 339/2,032 (16.7) |
| Iceland | 75 | 111 | 15 (20.0) | 27 (24.3) | 11/60 (18.3) | 30/111 (27.0) | 14/73 (19.2) | 38/109 (34.9) |
| Ireland | 2,289 | 2,083 | 539 (23.6) | 560 (26.9) | 308/1,936 (15.9) | 527/2,079 (25.3) | 436/2,233 (19.5) | 705/2,035 (34.6) |
| Italy* | 16,678 | 11,025 | 4,920 (29.5) | 3,356 (30.4) | 1,889/14,723 (12.8) | 2,391/10,979 (21.8) | 2,384/15,961 (14.9) | 2,237/10,672 (21.0) |
| Latvia | 723 | 252 | 108 (14.9) | 52 (20.6) | 127/616 (20.6) | 49/250 (19.6) | 68/667 (10.2) | 65/243 (26.7) |
| Lithuania | 614 | 370 | 59 (9.6) | 53 (14.3) | 81/519 (15.6) | 107/370 (28.9) | 38/585 (6.5) | 35/355 (9.9) |
| Luxembourg | 287 | 169 | 80 (27.9) | 46 (27.2) | 24/242 (9.9) | 33/167 (19.8) | 43/255 (16.9) | 49/160 (30.6) |
| Malta | 122 | 299 | 38 (31.1) | 85 (28.4) | 15/98 (15.3) | 59/299 (19.7) | 18/115 (15.7) | 93/287 (32.4) |
| Moldova | 123 | 498 | 18 (14.6) | 170 (34.1) (34.128.4) | 11/109 (10.1) | 119/498 (23.9) | 24/117 (20.5) | 150/474 (31.6) |
| Netherlands | 3,912 | 3,851 | 1,402 (35.8) (35.8) | 1,462 (38.0) | 673/3,326 (20.2) | 1,152/3,842 (30.0) | 1,117/3,799 (29.4) | 1,516/3,776 (40.1) |
| North Macedonia | 126 | 175 | 19 (15.1) | 33 (18.9) | 16/107 (15.0) | 44/175 (25.1) | 14/124 (11.3) | 21/165 (12.7) |
| Norway | 2,151 | 2,957 | 350 (16.3) | 556 (18.8) | 309/1,867 (16.6) | 736/2,948 (25.0) | 345/2,083 (16.6) | 864/2,869 (30.1) |
| Poland | 2,868 | 4,025 | 591 (20.6) | 902 (22.4) | 366/2,474 (14.8) | 897/3,996 (22.4) | 336/2,788 (12.1) | 800/3,894 (20.5) |
| Portugal | 5,386 | 2,555 | 1,077 (20.0) | 812 (31.8) | 587/4,865 (12.1) | 563/2,45 (22.1) | 627/4,962 (12.6) | 649/2,444 (26.6) |
| Romania | 2,451 | 2,002 | 489 (20.0) | 367 (18.3) | 376/2,127 (17.7) | 421/1,997 (21.1) | 397/2,268 (17.5) | 447/1,898 (23.6) |
| Russia | 5,258 | 6,247 | 1,016 (19.3) | 1,390 (22.3) | 813/4,717 (17.2) | 1,434/6,221 (23.1) | 970/5,083 (19.1) | 1,648/6,013 (27.4) |
| Serbia | 1,147 | 1,041 | 171 (14.9) | 176 (16.9) | 143/1,015 (14.1) | 220/1,032 (21.3) | 151/1,103 (13.7) | 124/998 (12.4) |
| Slovakia | 605 | 1,003 | 65 (10.7) | 133 (13.3) | 73/491 (14.9) | 192/999 (19.2) | 40/553 (7.2) | 128/952 (13.4) |
| Slovenia | 1,032 | 685 | 125 (12.1) | 100 (14.6) | 90/894 (10.1) | 119/685 (17.4) | 63/984 (6.4) | 175/669 (26.2) |
| Spain* | 13,730 | 10,652 | 4,227 (30.8) | 3,549 (33.3) | 1,561/12,228 (12.8) | 2,578/10,627 (24.3) | 2,217/12,933 (17.1) | 2,837/10,339 (27.4) |
| Sweden | 3,252 | 4,443 | 641 (19.7) | 801 (18.0) | 468/2,726 (17.2) | 1,003/4,430 (22.6) | 593/3,190 (18.6) | 978/4,297 (22.8) |
| Switzerland* | 5,180 | 3,383 | 1,629 (31.5) | 1,030 (30.5) | 595/4,390 (13.6) | 774/3,372 (23.0) | 734/4,821 (15.2) | 961/3,241 (29.7) |
| Turkey | 1,987 | 1,855 | 651 (32.8) | 625 (33.7) | 499/1,746 (28.6) | 680/1,851 (36.7) | 120/1,867 (6.4) | 368/1,778 (20.7) |
| Ukraine | 1,785 | 1,201 | 222 (12.4) | 245 (20.4) | 230/1,570 (14.6) | 231/1,196 (19.3) | 253/1,712 (14.8) | 307/1,159 (26.5) |
| United Kingdom | 18,432 | 11,889 | 5,539 (30.1) | 3,473 (29.2) | 3,022/15,766 (19.2) | 3,610/11,867 (30.4) | 4,288/18,082 (23.7) | 4,140/11,621 (35.6) |
| **Total** | **180,979** | **126,261** | **46,484 (25.7)** | **35,590 (28.2)** | **23,958/156,018 (15.4)** | **31,301/125,837 (24.9)** | **27,905/170,945 (16.3)** | **32,480/121,766 (26.7)** |

CAI, condomless anal intercourse. ^1^ This study includes 46 countries, with four European microstates included in neighbouring (Andorra, Liechtenstein) or surrounding (Monaco, San Marino) countries, and with Albania, Montenegro and Kosovo merged to form a region; this results in 40 country-like entities included in this table. ^2^ These questions had substantial numbers of missing answers which were excluded. *Including overseas territories/dependencies and/or a microstate. **^‡^**The designation of Kosovo is without prejudice to positions on status and is in line with UNSCR 1244/1999 and the International Court of Justice Opinion on the Kosovo declaration of independence.
